# Supplementary material for: New phiomorph rodents from the latest Eocene of Egypt, and the impact of Bayesian “clock”-based phylogenetic methods on estimates of basal hystricognath relationships and biochronology
Source: PeerJ. 2016 Mar 1;4:e1717. doi: 10.7717/peerj.1717 (PMC4782727; doi:10.7717/peerj.1717)
Supplement: Appendix S1 [file peerj-04-1717-s004.docx]

**Supporting Appendix 1**: Justification for priors set on ages of included taxa. Note that upper and lower limits of magnetochrons is based on the recently revised GPTS presented by Ogg (2012).

*Non-hystricognathous “ctenodactyloid” outgroups*

*Chapattimys* *wilsoni* and *Birbalomys* spp. (primarily based on *Birbalomys sondaari*) are from the upper part (“faunal zone VIII”) of the Subathu Formation in northwestern India (Kumar et al., 1997; Gupta & Kumar, 2015). This level is considered to be “early” Lutetian in age by Gupta & Kumar (2015), but no specific upper boundary is provided for this estimate; however this interpretation gains support from the fact that older *Birbalomys* specimens (*Birbalomys* cf. *sondaari*) have recently been discovered in the later Ypresian of India (Gupta & Kumar 2015). Rather than assign included species a prior that covers the entire Lutetian, which could recover unrealistically young age estimates, we divided the Lutetian in half based on its currently recognized boundaries of 41.2-47.8 on the Geological Time Scale 2012, leading to a uniform age prior of 44.5-47.8 Ma for a uniform “early” Lutetian prior.

*Anadianomys declivis* is from the Rencun Member in the lower part of the Heti Formation exposed in Henan, China (Tong, 1997), and is considered to fall within the Sharamurunian Asian Land Mammal Age based on its fauna (Russell & Zhai, 1987; Holroyd & Ciochon, 1994), an assessment that is supported by Appearance Event Ordination (Tsubamoto et al., 2004). Wang et al. (2007) consider the end of the Shuramurunian to be coincident with the end of the Bartonian (late middle Eocene), which is currently placed at 37.8 Ma on the Geological Time Scale 2012, but place the boundary between the Irdinmanhan and Shuramurunian Asian Land Mammal Ages at ~42.5, earlier than the currently recognized boundary between the Lutetian and Bartonian stages (41.2). Based on this combined evidence, we assigned *A. declivis* a uniform prior of 37.8-42.5 Ma.

*Yuomys cavioides* has been found in the Rencun Member in the lower part of the Heti Formation, as well as the Shara Murun and Jiyuan faunas (Russell and Zhai, 1987; Meng and McKenna, 1998; Bowen et al., 2002; Tsubamoto et al., 2004). All of these faunas are considered to fall within the Shuramurunian Asian Land Mammal Age; as with *Anadianomys declivis*, we accordingly restrict *Y. cavioides* to a uniform age prior of 37.8-42.5 Ma.

*Petrokozlovia notos* has been found in the Khaychin (II, III, IV) faunas on the Mongolian Plateau (Meng & McKenna, 1998; Bowen et al., 2002), which fall within the Irdinmanhan Asian Land Mammal Age (Tsubamoto et al., 2004). Wang et al. (2007) correlated the Arshantan-Irdinmanhan boundary with the Bridgerian-Uintan boundary of North America (~46 Ma), while the Irdinmanhan-Shuramurunian boundary was placed at ~42.5 Ma; accordingly we restrict *P. notos* to a uniform age prior of 42.5-46 Ma.

*Asian “baluchimyines”*

*Confinniumys sidiki* and *Ottomania proavita*, from Süngülü, Turkey (de Bruijn et al., 2003), have been interpreted as being of late Eocene or early Oligocene age based on comparison of these and other species (dipodids and murids) with roughly contemporaneous African and Asian species, but are poorly constrained due to the highly endemic nature of the fauna. We conservatively place these species within the late Eocene-early Oligocene interval based on the current boundaries in the Geological Time Scale 2012 with a wide uniform prior (i.e., 28.1-37.8 Ma).

“Baluchimyines” from the Bugti Hills and the Zinda Pir Dome in Pakistan that were scored in this analysis — i.e., *Baluchimys*, *Bugtimys*, *Hodsahibia*, *Lindsaya*, and *Lophibaluchia* (Flynn et al., 1986; Marivaux et al., 2002; Marivaux & Welcomme, 2003) — all come from the lower part of the Chitarwata Formation (Lindsay et al., 2005; Metais et al., 2009) and are of contentious age. They were originally described as being of early Miocene age (Flynn et al., 1986), but are now universally considered to be of Oligocene age (Lindsay et al., 2005; Metais et al., 2009), though there is still debate about whether they are early Oligocene or late Oligocene in age. Two different interpretations of the magnetostratigraphic evidence proposed by Lindsay et al. (2005) suggest that the base of the formation could fall within Chron 11n.1r, or Chron 7Ar; i.e. either in the early Oligocene or late Oligocene. With this uncertainty in mind, we applied a wide uniform prior for the entirety of the Oligocene for these species (23-33.9 Ma).

*Baluchimys krabiense*, from the Bang Mark pit in the Krabi coal mine of Thailand (Marivaux et al., 2000), has been correlated with either Chron 12r or 13r (Benammi et al., 2001), the current boundaries of which form our uniform prior for this species (31 to 35 Ma).

Though the species has been found at several localities, the best-figured and described material of *Tsaganomys altaicus* from the Hsanda Gol Formation in Mongolia (Bryant & McKenna, 1995; Wang, 2001) is considered to be of early Oligocene age and was recovered from below a basalt that has been dated to ~31.5 Ma. We follow Kraatz & Geisler (2010) in considering these lower levels of the Hsanda Gol Formation to correlate with Chron 12r of the GPTS (31.03-33.16), and we use this range as the uniform prior on the appearance of *T*. *altaicus*’ diagnostic morphology as scored in our matrix.

*Paleogene African and South American hystricognaths*

*Canaanimys maquiensis* was recovered from the top of the Yahuarango Formation at the CTA-27 locality at Loreto in Peru (Antoine et al., 2012), and the authors employed ^40^Ar/^39^Ar dating and mammalian biochronology to narrow the age estimate for the locality to 41.6-40.94 Ma, which we employ as the bounds of our uniform prior for this species.

“*Protophiomys*” *tunisiensis* has been found at the Djebel el Kébar locality in central Tunisia. Marivaux et al. (2014) reported that glauconite grains on the fossils from Djebel el Kébar returned K-Ar ages ranging in age from 38.7±1.0 and 40.7±1.0. We place a broad uniform prior on this species that takes into account the uncertainty in these K-Ar dates, ranging from 37.7 to 41.7 Ma.

*Protophiomys algeriensis* has been found at the Bir el-Ater or Nementcha locality in northern Algeria (Coiffait et al., 1984; Jaeger et al., 1985), which is dated entirely on the basis of its mammalian fossils. There is now a general consensus that Bir el-Ater is probably either late Eocene (Priabonian) or late middle Eocene (Bartonian) in age (Seiffert, 2010; Coster et al., 2012; Marivaux et al., 2014), and the upper and lower boundaries of these stages delimit the uniform prior for this species (33.9-41.2 Ma).

Multiple species from two localities (DT-1 [“*Phiomys*” *hammudai*, “*Protophiomys*” *durattalahensis*, and *Talahphiomys libycus*) and DT-2 (“*Protophiomys*” aff. *durattalahensis*, *Talahphiomys lavocati*)] have been found along the Dur at-Talah Escarpment in central Libya (Jaeger et al., 2010), in beds that have been called the Idam Unit (Wight, 1980) or the Bioturbated Unit (Jaeger et al., 2010). Prior to the description of the species from DT-1 and -2, the Idam Unit mammals were most recently interpreted as possibly being intermediate in age between Quarries L-41 and A/B in the Fayum succession, putting them very close to the Eocene-Oligocene boundary (Seiffert, 2010). Jaeger et al. (2010) presented new magnetostratigraphic evidence which showed that the entire sampled zone of the Dur at-Talah Escarpment is of normal polarity, but they favored a correlation of this normal polarity zone escarpment with C18n.1n, which would put those sites in the late middle Eocene (Bartonian). Sallam et al. (2012) and Antoine et al. (2012) have since favored an age intermediate between the Fayum localities BQ-2 and L-41, a zone that is, like the Dur at-Talah Escarpment, also of entirely normal polarity (Seiffert et al., 2005; Seiffert, 2006). To take into account the great uncertainty in the age of DT-1 and DT-2 reflected in these various interpretations, we set a wide uniform prior that spanned from the oldest possible age given the magnetostratigraphic correlation proposed by Jaeger et al. (2010) (39.6 Ma) to the younger bound of the Chron in which the only rodent species that Jaeger et al. (2010) consider to be shared with the Fayum succession (*Talahphiomys lavocati*) occurs (i.e., Fayum Quarry E), given Seiffert’s (2006) preferred magnetostratigraphic correlation (31 Ma).

*Turkanamys hexalophus* has been found solely at the LOK 13 locality at Lokone Hill, in the Lokone Sandstone Formation that is exposed in the Turkana Basin of northern Kenya (Ducrocq et al., 2010; Marivaux et al., 2012). The age of the site is constrained entirely by mammalian biochronology; based on available evidence Ducrocq et al. (2010) suggested a late Oligocene age, and this estimate was followed by Marivaux et al. (2012). Given the ambiguity of the evidence that has been presented thus far, however, we considered it preferable to assign a broad uniform prior that encompassed the entire Oligocene (i.e., 23-33.9 Ma).

The Fayum succession provides important temporal control on this analysis because it includes several rodent faunas of various ages (Wood, 1968; Holroyd, 1994; Sallam et al., 2009, 2011, 2012), all of which have been tied into a single magnetostratigraphic column (Kappelman et al., 1992; Seiffert et al., 2005, 2008; Seiffert, 2006). Here we employ the magnetostratigraphic correlation that was proposed by Seiffert (2006), and use the upper and lower bounds of each chron to delimit the uniform priors for each species. Species from Locality BQ-2 (*Protophiomys aegyptensis* and *Waslamys attiai*) fall into a zone of normal polarity that Seiffert et al. (2005) and Seiffert (2006) correlated with Chron 17n.1n, the boundaries of which are now 36.7-37.8 Ma. As noted by Seiffert (2006), correlation of the zone of reversed polarity sampled at Quarry L-41 with Chron 13r of the GPTS would not rule out the possibility of an earliest Oligocene age (because Chron 13r is largely late Eocene but does cross the Eocene-Oligocene boundary), but he argued that a major unconformity just above the locality was likely due to near-coastal erosion associated with the major marine regression that occurred near the Eocene-Oligocene boundary. For this reason we have set a uniform prior for L-41 species (*Acritophiomys bowni*, *Birkamys korai*, *Gaudeamus aslius*, *Gaudeamus hylaeus*, *Mubhamys vadumensis*) that extends from the base of Chron 13r to the Eocene-Oligocene boundary (35 Ma to 33.9 Ma), while *Gaudeamus aegyptius* and *Phiomys andrewsi* from Quarry A and Quarry B respectively (probably from the same zone of reversed polarity as that documented at L-41, as there are no intervening samples of normal polarity) are assigned a uniform prior that is post-Eocene (33.9) but before the termination of Chron 13r (33.7 Ma). Both *Gaudeamus aegyptius* and *Phiomys andrewsi* have also been identified at Quarry E (Holroyd, 1994), but note that their extensions to this younger level do not reflect age uncertainty but rather range extension, and in this case we are most concerned about the time by which the diagnostic morphology of *G. aegyptius* and *P. andrewsi* (as scored in this matrix) had appeared, not how long it persisted. Regarding other species, *Neophiomys paraphiomyoides* from Quarry G was placed in Chron 12n (30.6-31 Ma); “*Paraphiomys*” *simonsi* is only known from Quarries I and M, *Phiocricetomys minutus* is only found at Quarry I, and the definitive *Metaphiomys beadnelli* specimens that were scored for this matrix are based on specimens from I and M — both Quarries I and M fall within a zone or normal polarity that Seiffert (2006) correlated with Chron 11r (29.2-30 Ma); and “*Phiomys*” *lavocati* makes its first definitive appearance in the Fayum succession at Quarry E, which falls within Chron 12r (31-33.2 Ma) given Seiffert’s preferred correlation.

*Neogene African hystricognaths*

*Diamantomys luederitzi* has been found at several sites in east Africa and Namibia (e.g., Lavocat, 1973; Mein & Pickford, 2008), with the more complete specimens on which scoring is based having been found at Songhor in western Kenya. The fossils from Songhor are likely no older than 20 Ma (Pickford & Andrews, 1986; Cote et al., 2014), but fossils from Namibia are not as well constrained (Mein & Pickford 2008). We place a uniform prior on age of 19-21 Ma to partially account for this uncertainty.

Scorings for *Paraphiomys* spp. are based largely on specimens of *Paraphiomys pigotti* from Rusinga Island in western Kenya (Lavocat, 1973). This species has been found in both the Hiwegi and Kulu Formations on Rusinga (Lavocat, 1973; Peppe et al., 2009), based on available evidence these sites likely range in age from 15-17.8 Ma (Peppe et al., 2009), which we use as our uniform prior.

The age range of middle Miocene *Paraulacodus indicus*, from the Siwaliks of Pakistan, has been estimated by Flynn and Winkler (1994) as being 12.5-12.9 Ma, and we use these bounds as the limits of our uniform age prior for this species.

References for Supporting Appendix 1

Antoine P-O, Marivaux L, Croft DA, Billet G, Ganerød M, Jaramillo C, Martin T, Orliac MJ, Tejada J, Altamirano AJ, Duranthon F, Fanjat G, Rousse S, Gismondi RS. 2012. Middle Eocene rodents from Peruvian Amazonia reveal the pattern and timing of caviomorph origins and biogeography. Proceedings of the Royal Society B: Biological Sciences 279:1319–1326.

Benammi M, Chaimanee Y, Jaeger J-J, Suteethorn V, Ducrocq S. 2001. Eocene Krabi Basin (southern Thailand): Paleontology and magnetostratigraphy. Geological Society of America Bulletin 113:265–73.

Bowen GJ, Clyde WC, Koch PL, Ting SY, Alroy J, Tsubamoto T,Wang YQ,Wang Y. 2002. Mammalian dispersal at the Paleocene-Eocene boundary. Science 295: 2062–2065.

de Bruijn H, Ünay E, Saraç G, Yïlmaz A. 2003. A rodent assemblage from the Eo/Oligocene boundary interval near Süngülü, Lesser Caucasus, Turkey. Coloquios de Paleontología vol Ext 1: 47–76.

Bryant JD, McKenna MC. 1995. Cranial anatomy and phylogenetic position of *Tsaganomys altaicus* (Mammalia: Rodentia) from the Hsanda Gol Formation (Oligocene), Mongolia. American Museum Novitates 3156:1–15.

Coiffait P-É, Coiffait B, Jaeger J-J, Mahboubi M. 1984. Un nouveau gisement à mammifères fossiles d'âge Éocène supérieur sur le versant sud des Nementcha (Algérie orientale): découverte des plus anciens rongeurs d'Afrique. Comptes Rendus de l’Académie des Sciences, Serie II 299:893-898.

Coster P, Benammi M, Mahboubi M, Tabuce R, Adaci M, Marivaux L, Bensalah M, Mahboubi S, Mahboubi A, Mebrouk F, Maameri C, Jaeger J-J. 2012. Chronology of the Eocene continental deposits of Africa: Magnetostratigraphy and biostratigraphy of the El Kohol and Glib Zegdou Formations, Algeria. GSA Bulletin 124:1590-1606.

Cote S, Malit N, Nengo I. 2014. Additional mandibles of *Rangwapithecus gordoni*, an early Miocene catarrhine from the Tinderet localities of Western Kenya. American Journal of Physical Anthropology 153:341–352.

Ducrocq S, Boisserie J-R, Tiercelin J-J, Delmer C, Garcia G, Manthi FK, Leakey MG, Marivaux L, Otero O, Peigné S, Tassy P, Lihoreau F. 2010. New Oligocene vertebrate localities from northern Kenya (Turkana Basin). Journal of Vertebrate Paleontology 30:293-299.

Flynn LJ, Winkler AJ. 1994. Dispersalist implications of *Paraulacodus indicus*: a South Asian rodent of African affinity. Historical Biology 9:223–235.

Flynn LJ, Jacobs LL, Cheema IU. 1986. Baluchimyinae, a new ctenodactyloid rodent subfamily from the Miocene of Baluchistan. American Museum Novitates 2841: 1–31.

Gupta S, Kumar K. 2015. Early Eocene rodents (Mammalia) from the Subathu Formation of type area (Himachal Pradesh), NW sub–Himalaya, India: Palaeobiogeographic implications. Journal of Earth System Science 124: 1201–1221.

Holroyd PA. 1994. An Examination of Dispersal Origins for Fayum Mammalia. Ph.D. dissertation, Duke University, Durham, North Carolina.

Holroyd P, Ciochon RL. 1994. Relative ages of Eocene primate–bearing deposits of Asia. In Kay RF, Fleagle JG, eds. Anthropoid Origins. New York: Plenum Press, pp. 123–141.

Jaeger J-J, Denys C, Coiffait B. 1985. New Phiomorpha and Anomaluridae from the late Eocene of north-west Africa: Phylogenetic implications. In Luckett WP, Hartenberger J-L, eds. Evolutionary Relationships among Rodents - A Multidisciplinary Analysis. New York: Plenum Press, pp. 567-588.

Jaeger J-J, Marivaux L, Salem M, Bilal AA, Benammi M, Chaimanee Y, Duringer P, Marandat B, Metais E, Schuster M, Valentin X, Brunet M. 2010. New rodent assemblages from the Eocene Dur at-Talah escarpment (Sahara of central Libya): systematic, biochronological, and palaeobiogeographical implications. Zoological Journal of the Linnean Society 160:195-213.

Kappelman J, Simons EL, Swisher CC III. 1992. New age determinations for the Eocene-Oligocene boundary sediments in the Fayum Depression, northern Egypt. Journal of Geology 100:647-668.

Kraatz BP, Geisler JH. 2010. Eocene–Oligocene transition in Central Asia and its effects on mammalian evolution. Geology 38:111-114.

Kumar K, Srivastava R, Sahni A. 1997. Middle Eocene rodents from the Subathu Group, northwest Himalaya. Palaeovertebrata 26: 83–128.

Lavocat R. 1973. Les Rongeurs du Miocène d’Afrique Orientale, Miocène inférieur. Mémoires et Travaux de l’Institut de Montpellier de l’Ecole Pratique des Hautes Etudes 1:1-284.

Marivaux L, Welcomme J-L. 2003. Additional diatomyid and baluchimyine rodents from the Oligocene of Pakistan (Bugti Hills, Baluchistan): systematic and paleobiogeographic implications. Journal of Vertebrate Paleontology 23: 420–434.

Marivaux L, Benammi M, Ducrocq S, Jaeger J-J, Chaimanee Y. 2000. A new baluchimyine rodent from the Late Eocene of the Krabi Basin (Thailand): paleobiogeographic and biochronologic implications. Comptes Rendus de l’Académie des Sciences, Sciences de la Terre et des planètes 331: 427–433.

Marivaux L, Welcomme J-L, Vianey-Liaud M, Jaeger J-J. 2002. The role of Asia in the origin and diversification of hystricognathous rodents. Zoologica Scripta 31: 225–239.

Marivaux L, Lihoreau F, Manthi FK, Ducrocq S. 2012. A new basal phiomorph (Rodentia, Hystricognathi) from the late Oligocene of Lokone (Turkana Basin, Kenya). Journal of Vertebrate Paleontology 32:646-657.

Marivaux L, Essid E, Marzougui W, Ammar H, Adnet S, Marandat B, Merzeraud G, Tabuce R, Vianey-Liaud M. 2014. A new and primitive species of *Protophiomys* (Rodentia, Hystricognathi) from the late middle Eocene of Djebel el Kébar, Central Tunisia. Palaeovertebrata 38:e2.

Mein P, and Pickford M. 2008. Early Miocene Rodentia from the Northern Sperrgebiet, Namibia. Memoirs of the Geological Society of Namibia 20:235-290.

Meng J, McKenna MC. 1998. Faunal turnovers of Palaeogene mammals from the Mongolia Plateau. Nature 394: 364–367.

Métais G, Antoine P-O, Baqri SRH, Crochet J-Y, de Franceschi D, Marivaux L, Welcomme J-L. 2009. Lithofacies, depositional environments, regional biostratigraphy and age of the Chitarwata Formation in the Bugti Hills, Balochistan, Pakistan. Journal of Asian Earth Sciences 34:154–167.

Ogg JG. 2012. Geomagnetic Polarity Time Scale. In Gradstein F, Ogg JG, Schmitz M, and Ogg G, eds. The Geological Time Scale 2012. Amsterdam: Elsevier, pp. 85–114.

Peppe D, McNulty K, Cote S, Harcourt-Smith W, Dunsworth H, Van Couvering J. 2009. Stratigraphic interpretation of the Kulu Formation (Early Miocene, Rusinga Island, Kenya) and its implications for primate evolution. Journal Human Evolution 56:447-461.

Pickford M, Andrews P. 1981. The Tinderet Miocene sequence in Kenya. Journal of Human Evolution 10:11-33.

Pickford M, Senut B, Morales J, Mein P, Sánchez IM. 2008. Mammalia from the Lutetian of Namibia. Memoirs of the Geological Society of Namibia 20:465-514.

Russell DE, Zhai R. 1987. The Paleogene of Asia: mammals and stratigraphy. Mémoires du Muséum national d’Histoire naturelle (Série C Sciences de la Terre) 52: 1–488.

Sallam HM, Seiffert ER, Steiper ME, Simons EL. 2009. Fossil and molecular evidence constrain scenarios for the early evolutionary and biogeographic history of hystricognathous rodents. Proceedings of the National Academy of Sciences, USA 106: 16722–16727.

Sallam HM, Seiffert ER, Simons EL. 2011. Craniodental morphology and systematics of a new family of hystricognathous rodents (Gaudeamuridae) from the late Eocene and early Oligocene of Egypt. PLoS ONE 6:e16525.

Sallam HM, Seiffert ER, Simons EL. 2012. A basal phiomorph (Rodentia, Hystricognathi) from the late Eocene of the Fayum Depression, Egypt. Swiss Journal of Palaeontology 131:283-301.

Seiffert ER. 2006. Revised age estimates for the later Paleogene mammal faunas of Egypt and Oman. Proceedings of the National Academy of Sciences, USA 103:5000–5005.

Seiffert ER. 2010. Chronology of Paleogene mammal localities. In Werdelin L, Sanders WJ, eds. Cenozoic Mammals of Africa. Berkeley: University of California Press, pp. 19-26.

Seiffert ER, Simons EL, Clyde WC, Rossie JB, Attia Y, Bown TM, Chatrath P, Mathison M. 2005. Basal anthropoids from Egypt and the antiquity of Africa's higher primate radiation. Science 310:300-304.

Seiffert ER, Bown TM, Clyde WC, Simons EL. 2008. Geology, paleoenvironment, and age of Birket Qarun Locality 2 (BQ-2), Fayum Depression, Egypt. In Fleagle JG, Gilbert CC, eds. Elwyn L Simons: A Search for Origins. New York: Springer, pp. 71-86.

Tong Y. 1997. Middle Eocene small mammals from Liguanqiao Basin of Henan Province and Yuanqu Basin of Shanxi Province, central China. Palaeontologia Sinica (Ser. C) 26: 1–256.

Tsubamoto T, Takai M, Egi N. 2004. Quantitative analyses of biogeography and faunal evolution of middle to late Eocene mammals in east Asia. Journal of Vertebrate Paleontology 24: 657–667.

Wang B. 2001. On Tsaganomyidae (Rodentia, Mammalia) of Asia. American Museum Novitates 3317: 1-50.

Wang Y, Meng J, Ni X, Li C. 2007. Major events of Paleogene mammal radiation in China. Geological Journal 42: 415–430.

Wight AWR. 1980. Palaeogene vertebrate fauna and regressive sediments of Dur at Talhah, Southern Sirt Basin, Libya. In Salem MJ, Busrewil MT, eds. Geology of Libya, Volume I. San Diego: Academic Press, pp. 309-325.

Wood AE. 1968. Early Cenozoic mammalian faunas, Fayum Province, Egypt, Part II: the African Oligocene Rodentia. Peabody Museum Bulletin 28:23-205.
