# Supplementary figures and images for: New phiomorph rodents from the latest Eocene of Egypt, and the impact of Bayesian “clock”-based phylogenetic methods on estimates of basal hystricognath relationships and biochronology
Source: PeerJ. 2016 Mar 1;4:e1717. doi: 10.7717/peerj.1717 (PMC4782727; doi:10.7717/peerj.1717)

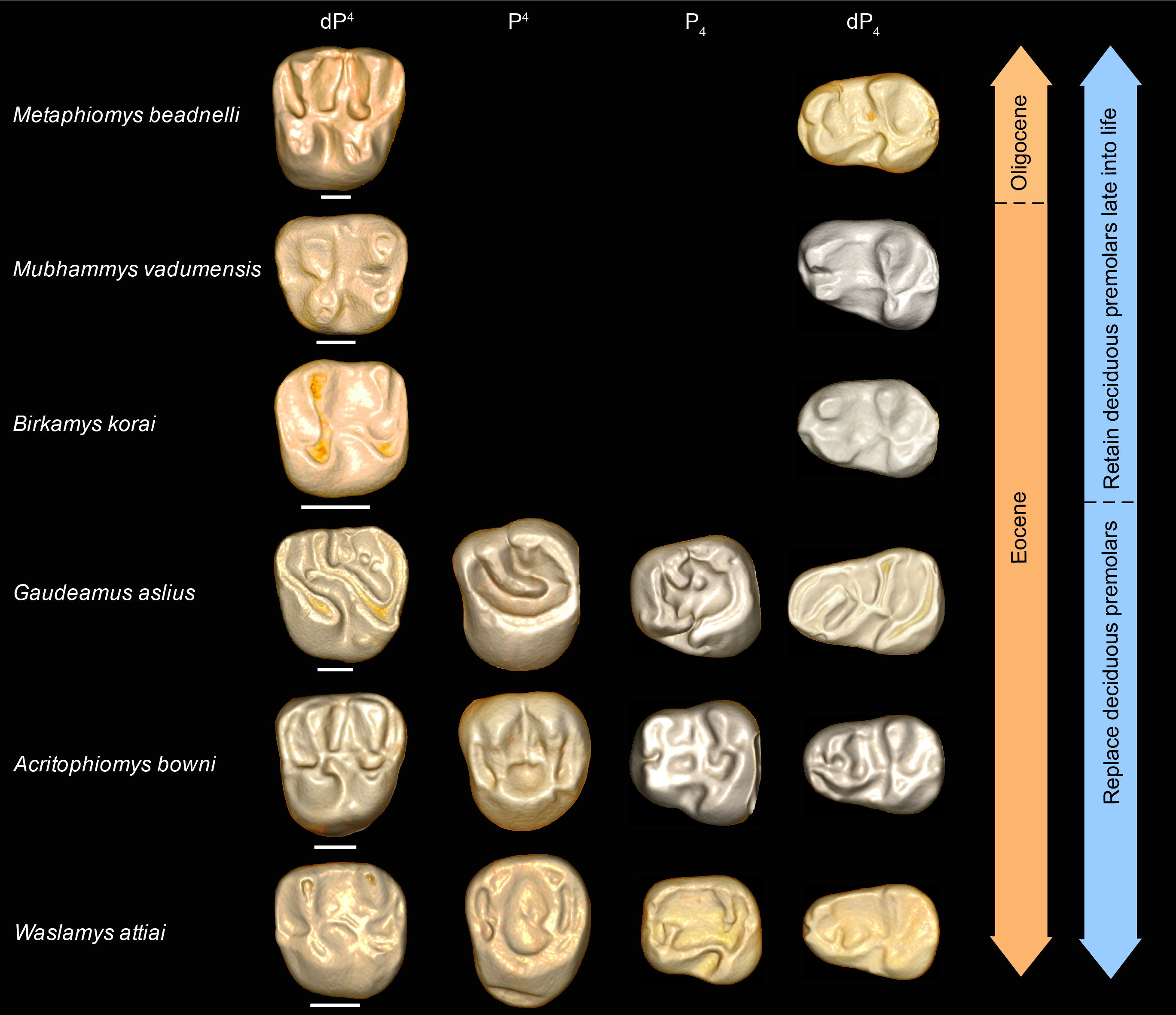

Supplement: Figure S1 — Metaphiomys beadnelli, dP4 (DPC 2431, reversed) and dP4 (DPC 6047, reversed); Mubhammys vadumensis, dP4 (DPC 14324) and dP4 (DPC 13220); Birkamys korai, dP4 (DPC 17457, reversed) and dP4 (DPC 22737, reversed); Gaudeamus aslius, dP4 (DPC 15748, reversed), P4 (CGM 66006, reversed), P4 (DPC 17677, reversed), and dP4 (DPC 16920); Acritophiomys bowni, dP4 (DPC 12662, reversed), P4 (CGM 83705, reversed), P4 (DPC 14168), and dP4 (DPC 20288, reversed); Waslamys attiai, dP4 (DPC 21365E), P4 (DPC 23305G, reversed), P4 (DPC 21371N), and dP4 (DPC 21293P). [file peerj-04-1717-s006.png]
